# Validation of the ABC Method for Gastric Cancer Risk Stratification Across *Helicobacter pylori* Infections With Diverse CagA Status and Subtypes in Brazil

**DOI:** 10.1002/cam4.71016

**Published:** 2025-06-27

**Authors:** Luis Masuo Maruta, Asuka Furukawa, Heinrich Bender Kohnert Seidler, Aloisio Felipe‐Silva, Keisuke Uchida, Daisuke Kobayashi, Kurara Yamamoto, Junko Minami, Masaki Sekine, Minako Takagi, Renato Takayuki Hassegawa, Eduardo Koji Marchi Ogawa, Rodrigo Barbosa Villaça, Tecio de Araujo Couto, Jorge Alberto Capra Biasuz, Edmar Tafner, Ana Luiza Werneck‐Silva, Simone Perez Pilli, José Guilherme Nogueira da Silva, Leonard Medeiros da Silva, Ricardo Ambrosio Fock, Chinatsu Ogura, Yumi Mizuguchi, Keiko Miura, Kouhei Yamamoto, Yoshinobu Eishi, Kenichi Ohashi

**Affiliations:** ^1^ Hospital Universitário, Endoscopy Service Universidade de São Paulo (USP) São Paulo SP Brazil; ^2^ Hospital Japonês Santa Cruz São Paulo SP Brazil; ^3^ Department of Human Pathology, Graduate School of Medical and Dental Sciences Institute of Science Tokyo (Formerly Known as Tokyo Medical and Dental University) Tokyo Japan; ^4^ Laboratório Brasiliense Brasília Distrito Federal Brazil; ^5^ Hospital Universitário, Anatomic Pathology Service Universidade de São Paulo (USP) São Paulo SP Brazil; ^6^ Division of Surgical Pathology Institute of Science Tokyo Hospital (Formerly Known as Tokyo Medical and Dental University Hospital) Tokyo Japan; ^7^ Clinica Brasília Brasília Distrito Federal Brazil; ^8^ Faculty of Pharmaceutical Sciences Universidade de São Paulo (USP) São Paulo SP Brazil

**Keywords:** ABC method, CagA subtyping, gastric cancer risk stratification, *Helicobacter pylori* CagA diversity, immunohistochemistry, intestinal metaplasia, intrafamilial transmission, noninvasive screening, OLGA/OLGIM staging, serologic biomarkers

## Abstract

**Background:**

Gastric cancer survival rates vary across countries due to differences in access to early diagnostic testing and healthcare quality. Endoscopy, though accurate, is not feasible for mass screening. The ABC method, which combines serum *Helicobacter pylori* (Hp) antibody and pepsinogen tests, has shown promise for gastric cancer risk stratification in Japan. However, its applicability in populations with diverse CagA status and subtypes remains uncertain.

**Materials and Methods:**

This prospective study in Brazil evaluated the performance of the ABC method in an endoscopy‐referred cohort with a heterogeneous distribution of Hp CagA status and subtypes. A recently validated immunohistochemical method was applied to formalin‐fixed paraffin‐embedded gastric biopsy samples to assess Hp infection, CagA expression, and CagA subtypes. Gastric pathology was evaluated using the updated Sydney System and OLGA/OLGIM staging and correlated with serum Hp antibody and pepsinogen levels in 586 patients, including 122 Japanese Brazilians.

**Results:**

Immunohistochemistry achieved a 98% success rate (577/586). The prevalence of Hp infection was 48%, with Western‐type CagA(+) (26%) and CagA(−) (18%) strains predominating. East Asian‐type CagA(+) strains (4%) were observed primarily among Japanese Brazilians, particularly in second‐generation individuals. Gastric pathology and serum markers differed significantly across CagA status and subtypes. Despite these differences, the ABC method's negative predictive values (NPVs) across all groups other than Group A (negative for both tests) remained high (97%/97% or 98%/100% for detecting OLGA/OLGIM stages ≥ II or ≥ III, and 94%/98% or 99%/100% for detecting antrum/corpus inflammation scores ≥ 2 or 3, respectively).

**Conclusions:**

These findings demonstrate the clinical relevance of CagA diversity for gastric cancer risk assessment. Although limited to an endoscopy‐referred cohort, the ABC method reliably identified low‐risk individuals (Group A) and may help reduce unnecessary endoscopies in screening programs, regardless of CagA status and subtypes. Broader, population‐based studies are needed to validate its generalizability and optimize its implementation.

## Graphical abstract

This prospective study in Brazil demonstrates the clinical relevance of CagA‐diverse *Helicobacter pylori* infections for gastric cancer risk assessment. The ABC method reliably identified low‐risk individuals (Group A) and may help reduce unnecessary endoscopies in screening programs, regardless of CagA status and subtypes.

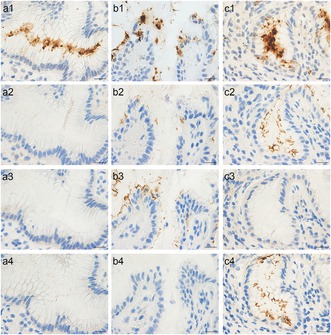

## 1 Introduction

Gastric cancer remains a major global health concern, responsible for approximately 800,000 deaths per year as of 2020 [1, 2]. Survival rates vary significantly across countries, with 5‐year survival rates of 20.6% in Brazil, 68.9% in Japan, and 60.3% in Korea [3]. This disparity may reflect differences in gastric cancer screening programs, access to early diagnostic testing, and healthcare infrastructure [3].

Endoscopy is the gold standard for gastric cancer detection, but its application in mass screening is limited by costs, workforce shortages, and restricted accessibility [4]. Therefore, noninvasive screening tools that stratify individuals based on gastric cancer risk could help optimize endoscopic referrals and improve early detection rates. One such tool is the serologic risk stratification approach known as the ABC method, a prescreening strategy used in Japan that measures serum levels of pepsinogens and *Helicobacter pylori* (Hp) antibodies [5, 6]. The ABC method has demonstrated cost‐effectiveness and high sensitivity for identifying individuals at elevated gastric cancer risk, potentially improving early detection rates compared to conventional mass screening [7, 8]. In Europe, efforts are underway to evaluate combinations of serologic markers, such as pepsinogens and Hp antibodies, in risk models for gastric cancer screening [9]. Similarly, a global review suggests that integrating serologic biomarkers into gastric cancer screening strategies may be particularly beneficial in countries with limited endoscopic capacity [10].

Although widely adopted in Japan, the effectiveness of the ABC method in populations with variation in Hp CagA status and subtypes remains unclear. In Japan, most Hp strains express East Asian‐type CagA with the EPIYA‐D motif [11], whereas in Brazil, the majority of strains carry Western‐type CagA with the EPIYA‐C motif, and a substantial proportion are CagA(−) [12]. Additionally, Japanese Brazilians may have greater exposure to East Asian‐type Hp, potentially influencing their gastric cancer risk [13]. CagA plays a critical role in promoting mucosal inflammation, atrophy, and intestinal metaplasia, which are precursors to diffuse‐ and intestinal‐type gastric cancers [14, 15]. Therefore, optimal cutoff values for pepsinogen and antibody tests in the ABC method may require adjustment based on Hp CagA status and subtypes.

CagA status and subtypes are traditionally evaluated by polymerase chain reaction (PCR) and sequencing of the EPIYA repeat region using DNA from Hp isolated from fresh gastric biopsy samples. We recently developed two monoclonal antibodies (mAbs) that specifically recognize either the EPIYA‐C or EPIYA‐D motifs of the CagA protein in formalin‐fixed paraffin‐embedded (FFPE) tissue sections [16], thereby enabling a novel immunohistochemical method for CagA subtyping. These newly developed mAbs, in combination with commercially available mAbs against Hp‐specific lipopolysaccharides and a conserved CagA region, allowed comprehensive characterization of Hp by immunohistochemistry (IHC) in FFPE tissue, including Hp infection status (positive or negative), CagA expression (positive or negative), and CagA subtypes (East Asian‐type or Western‐type) [16].

This prospective study in Brazil aimed to evaluate the utility of the ABC method for gastric cancer risk stratification in an endoscopy‐referred cohort with diverse Hp infection patterns. Using our novel immunohistochemical method, we classified Hp infections by CagA status and subtypes, and assessed their associations with gastric pathology and serum markers. We also explored potential familial clustering of East Asian‐type infections among Japanese Brazilians. These findings may inform population‐specific adaptation of the ABC method in regions with heterogeneous Hp strains.

## 2 Materials and Methods

### 2.1 Patients and Samples

Patients undergoing gastric endoscopy for the evaluation of gastric lesions, with or without symptoms, were recruited from three hospitals (Hospital Universitário da Universidade de São Paulo (USP), Hospital Japonês Santa Cruz, and Clínica Brasília) between September 2020 and April 2022. Exclusion criteria included a history of Hp eradication therapy, prior gastrectomy, hepatorenal dysfunction, or lack of informed consent. Written informed consent was obtained from all patients. After excluding 12 patients diagnosed with tumors (9 with gastric cancer, 1 with gastric MALT lymphoma, 1 with gastric neuroendocrine tumor, and 1 with laryngeal carcinoma) and 7 cases with incomplete biopsy samples, a total of 586 paired serum and complete biopsy samples were analyzed. All patients had complete serum marker data with no missing values. The study protocol was approved by the ethics committees of Hospital Universitário da USP (HUUSP CEP76 CAAE 52744016.7.0000.0076) and Institute of Science Tokyo Hospital (formerly known as Tokyo Medical and Dental University Hospital; M2018‐244 and M2019‐072).

### 2.2 Serum Markers

Fasting blood samples were collected, and serum samples were stored at −20°C until analysis. Serum Hp antibody, pepsinogen I (PGI), and pepsinogen II (PGII) levels were measured using an automated analyzer (Cobas 6000, Roche, Basel, Switzerland) and latex agglutination test kits (L‐Z test; Eiken, Tokyo, Japan) at the central clinical laboratory of Hospital Universitário da USP in Brazil. Samples exceeding the assay's range were diluted and recalculated accordingly. Following the manufacturer's guidelines, serum samples with a PGI level ≤ 70 ng/mL and a PGI/II ratio ≤ 3.0 were considered positive for the pepsinogen test, and serum samples with an Hp antibody level ≥ 5 U/mL were considered positive for the Hp antibody test. Patients were categorized into four groups based on the results of the pepsinogen and Hp antibody tests following the ABC classification system proposed by Miki [5]: Group A: negative for both tests; Group B: pepsinogen test (−), Hp antibody test (+); Group C: positive for both tests; and Group D: pepsinogen test (+), Hp antibody test (−). For exploratory analysis, PGII levels ≥ 20 ng/mL were considered elevated and served as an independent marker.

### 2.3 Gastric Biopsy Samples

Five biopsy samples were collected from each patient during endoscopic examination: two from the corpus, one from the incisura angularis, and two from the antrum. After fixation in 10% neutral‐buffered formalin, the samples were embedded in a paraffin block, with each specimen arranged according to its anatomic location. The resulting FFPE blocks were examined at the surgical pathology laboratory of Institute of Science Tokyo Hospital (formerly known as Tokyo Medical and Dental University Hospital) in Japan. Histologic assessments, including grading scores (0–3 scale) for activity, inflammation, atrophy, and intestinal metaplasia in the antrum and corpus, were performed according to the updated Sydney System [17]. Two experienced pathologists (D.K. and K.Y.) independently evaluated the histology, and any discrepancies were resolved by consensus. Stages (0–IV) were determined using the Operative Link on Gastritis Assessment (OLGA) system [18], or the Operative Link on Gastric Intestinal Metaplasia (OLGIM) Assessment system [19], based on the grading scores for atrophy or intestinal metaplasia in the antrum and corpus, respectively. Histologically unfavorable findings were defined as Sydney System grading scores ≥ 1 or OLGA/OLGIM stages ≥ I. The patients' clinicopathologic characteristics—including age, sex, ethnicity, symptoms, Kimura‐Takemoto classification [20], OLGA/OLGIM classification, and Hp infection status determined by Giemsa staining—are detailed in Table S1.

### 2.4 Immunohistochemistry

To determine Hp infection status, CagA expression, and CagA subtypes, IHC analysis was performed using four types of mAbs [16]: one targeting Hp lipopolysaccharides (anti‐Hp mAb; D369‐3, Medical and Biological Laboratories, Nagoya, Japan), one targeting a conserved region of the CagA protein (anti‐CagA mAb; sc‐28368, Santa Cruz Biotechnology, Dallas, TX, USA), and two targeting for the EPIYA‐C and EPIYA‐D motifs of the CagA protein, respectively. Serial sections (4 μm thick) were processed using a Leica BOND‐III automated system (Leica Microsystems Inc., Tokyo, Japan). Detection was performed using the BOND Polymer Refine Detection kit (#DS9800, Leica Microsystems Inc.). The staining procedure included deparaffinization, peroxidase blocking, antigen retrieval, incubation with primary antibodies at room temperature for 15 min, and counterstaining, in accordance with the manufacturer's instructions. IHC positivity was defined as one or more distinct clusters of dot‐like signals on the surface of gastric foveolar epithelium in at least one of five biopsy samples.

### 2.5 PCR

A 10‐μm‐thick tissue section from each FFPE gastric biopsy block was placed in a sterile 1.5‐mL microcentrifuge tube. DNA was extracted from each section using TaKaRa DEXPAT (9091, Takara Bio, Shiga, Japan), and purified with Ethachinmate (318‐01793, Nippon Gene, Tokyo, Japan) following the manufacturer's instructions. Real‐time PCR was performed to amplify bacterial 16S rRNA, the *cagA* gene, and the EPIYA‐C and EPIYA‐D motifs using previously described primers and probes [16]. The reaction mixture contained 5 μL of template DNA, primers, probe, and 2× SensiFAST Probe Hi‐ROX Mix (BIO‐82020, BIOLINE, USA) in a total volume of 50 μL. Amplification was carried out using the ABI PRIZM 7900HT system (Applied Biosystems, Foster City, CA, USA) under standard conditions. Serial dilutions of bacterial DNA were used as references for quantification, and negative controls (lacking bacterial DNA) were included. PCR results were considered positive for each target if amplification of any specific bacterial genomic fragment was detected.

### 2.6 Statistical Analysis

Associations between two discrete variables were assessed using odds ratios (ORs) with 95% confidence intervals (95% CI) and Fisher's exact test. Differences in serum marker levels (PGI, PGII, Hp antibody, and PGI/II ratios) between groups were evaluated using the Mann–Whitney *U* test. Relationships between serum markers and gastric pathology were analyzed by calculating the area under the curve (AUC) from receiver operating characteristic (ROC) curves for each group defined by CagA status and subtypes. Cutoff values for PGI, Hp antibody, and the PGI/II ratio were initially based on the manufacturer's recommendations for clinical reference. For exploratory analyzes, cutoff values were determined via ROC curve analysis, using the Youden Index to maximize both sensitivity and specificity. Gastric pathology scores and stages among the three ABC classification groups were compared using the Kruskal–Wallis test followed by Dunn's multiple comparison test. All statistical analyzes were performed using R (version 4.3.2), and *p*‐values < 0.05 were considered statistically significant.

## 3 Results

### 3.1 Hp Classification by IHC

**FIGURE 1:**
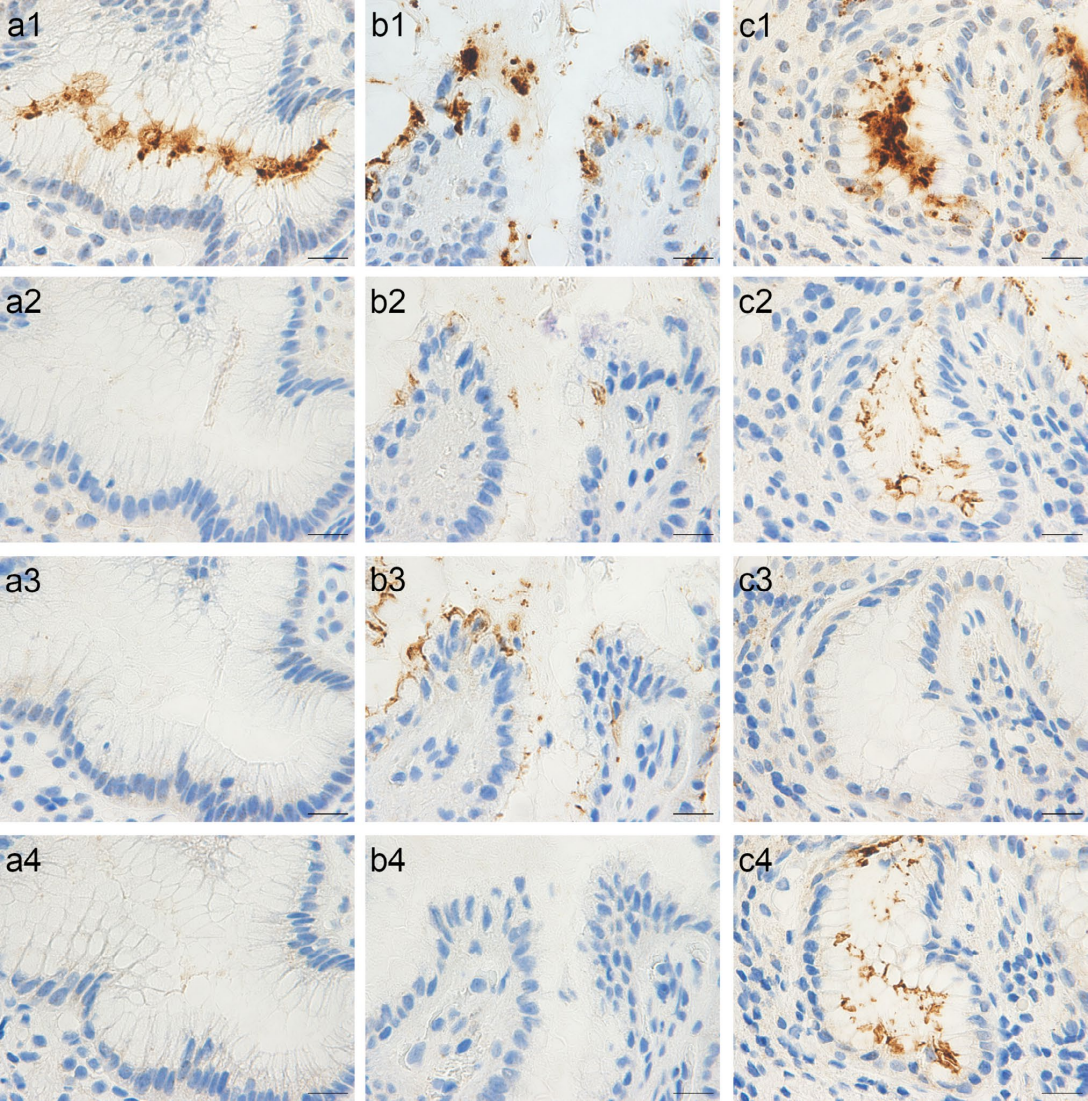
Immunohistochemical classification of CagA status and subtypes of *Helicobacter pylori* (Hp) in gastric biopsy samples. Representative paired images are shown for each CagA status and subtype confirmed by PCR: CagA(−) (a), Western type (b), and East Asian type (c). Staining was performed using monoclonal antibodies specific for Hp (1), CagA (2), EPIYA‐C (3), and EPIYA‐D (4). Scale bars: 20 μm.

Representative IHC findings are shown in Figure 1. Hp infection was classified into four groups based on Hp infection status, CagA expression, and CagA subtypes: negative for infection [Hp(−)/CagA(−)/EPIYA‐C(−)/EPIYA‐D(−)]; CagA(−) infection [Hp(+)/CagA(−)/EPIYA‐C(−)/EPIYA‐D(−)]; Western‐type CagA(+) infection [Hp(+)/CagA(+)/EPIYA‐C(+)/EPIYA‐D(−)]; and East Asian‐type CagA(+) infection [Hp(+)/CagA(+)/EPIYA‐C(−)/EPIYA‐D(+)].

Among the 586 gastric biopsy samples analyzed, IHC‐based Hp classification was successful in 577 cases (98%), yielding the following distribution: 302 cases (52%) were negative for infection, 102 cases (18%) had CagA(−) infection, 151 cases (26%) had Western‐type infection, and 22 cases (4%) had East Asian‐type infection. CagA classification was inconclusive in nine cases: six showed Hp(+)/CagA(+)/EPIYA‐C(−)/EPIYA‐D(−), and three showed Hp(+)/CagA(−)/EPIYA‐C(+)/EPIYA‐D(−).

IHC and PCR results were concordant in 472 (82%) of the 577 successfully classified cases. The distribution in these concordant cases was as follows: 283 (60%) were negative for infection, 71 (15%) had CagA(−) infection, 100 (21%) had Western‐type infection, and 18 (4%) had East Asian‐type infection (Table S2). Clinicopathologic characteristics did not significantly differ between patients with successful classification by IHC and those with concordant classification by IHC and PCR (Table S1).

### 3.2 Familial Clustering of Hp Infection

Among the 577 biopsy samples with successful IHC‐based Hp classification, 122 (21%) were from Japanese Brazilians (Table 1). In this subgroup, 84 (69%) were negative for infection, 9 (7%) had CagA(−) infection, 9 (7%) had Western‐type infection, and 20 (16%) had East Asian‐type infection. East Asian‐type infection was detected in 22 patients (4% of the total) and was predominantly observed in Japanese Brazilians, with the exception of one patient of Korean descent and one of Chinese descent.

**TABLE 1:** Prevalence of *Helicobacter pylori* (Hp) infection, and CagA status and subtypes among 122 Japanese Brazilians within a cohort of 577 patients classified by immunohistochemistry.

	Hp(−)	CagA(−)	Western‐type	East Asian‐type	Total
Total number of cases	84	9	9	20^a^	122
Number of cases in the					
1st generation	5	0	0	1	6
2nd generation	48	6	2	12	68
3rd generation	23	2	6	1	32
4th generation	2	0	0	0	2
2nd‐generation half	4	0	1	6^b^	11
3rd‐generation half	2	1	0	0	3

^a^ A total of 22 cases of East Asian‐type infection were identified, including one Korean and one Chinese patient.

^b^ Among the six second half‐generation individuals (either parent Japanese), four had a Japanese father and two had a Japanese mother.

Notably, East Asian‐type infection was identified in 18 of 27 Hp‐infected Japanese Brazilians of the second generation (both parents Japanese) and the second half‐generation (either parent Japanese), suggesting a pattern of familial clustering, which may indicate intrafamilial transmission. It is important to note, however, that nearly half (18/38, 47%) of Hp‐infected Japanese Brazilians harbored Western‐type or CagA(−) Hp strains, demonstrating that not all cases conform to an expected pattern of vertical transmission.

The prevalence of East Asian‐type infection among Hp‐infected Japanese Brazilians was highest in the first‐generation (1/1, 100%). In the second‐generation individuals, the prevalence remained elevated (18/27, 67%) but declined markedly in the third‐ and fourth‐generation individuals (1/10, 10%). Among the six patients in the second half‐generation with East Asian‐type Hp infection, four had a Japanese father and two had a Japanese mother.

### 3.3 Hp Classification and Gastric Pathology

Gastric pathology was compared among groups defined by Hp infection status, CagA status, and CagA subtypes, using Sydney System scores and OLGA/OLGIM stages from 577 biopsy samples with successful IHC‐based classification. The prevalence of scores ≥ 1 or stages ≥ I was generally higher in infected than in noninfected patients, except for corpus intestinal metaplasia and OLGIM. Patients with CagA(+) infection more commonly had antrum/corpus atrophy and intestinal metaplasia scores ≥ 1 and OLGA/OLGIM stages ≥ I than patients with CagA(−) infection (ORs: 3.4, 4.5, and 4.0 for atrophy; 12.0, not applicable, and 13.6 for intestinal metaplasia, respectively) (Table 2). Activity and inflammation in the corpus also differed significantly between patients with CagA(+) and CagA(−) infections (ORs: 7.5 and 3.7, respectively), but not in the antrum. Antral intestinal metaplasia ≥ 1 and OLGIM ≥ I were significantly more prevalent in patients with East Asian‐type infection than in those with Western‐type infection (ORs: 3.5 and 3.7, respectively). These findings were similar to those observed in the 472 patients with concordant IHC and PCR classification (Table S3).

**TABLE 2:** Comparison of gastric pathology scores and stages among 577 patients grouped by CagA status and subtype, as determined by immunohistochemical classification.

		CagA(+) versus CagA(−) infection				East Asian‐type versus Western‐type infection			
		CagA (+)	CagA (−)	Odds (95% CI)	*p* *	East Asian	Western	Odds (95% CI)	*p* *
Antrum scores									
Activity	≥ 1	158	85	2.1	N.S.	18	140	0.4	N.S.
	0	15	17	(0.9–4.8)		4	11	(0.1–1.7)	
Inflammation	≥ 1	170	100	1.1	N.S.	22	148	—	N.S.
	0	3	2	(0.1–10)		0	3		
Atrophy	≥ 1	113	36	3.4	< 0.0001	17	96	1.9	N.S.
	0	60	66	(2.0–6.0)		5	55	(0.6–7.1)	
Metaplasia	≥ 1	57	4	12.0	< 0.0001	13	44	3.5	0.0075
	0	116	98	(4.2–47)		9	107	(1.3–10)	
Corpus scores									
Activity	≥ 1	149	46	7.5	< 0.0001	21	128	3.8	N.S.
	0	24	56	(4.1–14)		1	23	(0.6–63)	
Inflammation	≥ 1	167	90	3.7	0.0105	22	145	—	N.S.
	0	6	12	(1.2–12)		0	6		
Atrophy	≥ 1	61	11	4.5	< 0.0001	11	50	2.0	N.S.
	0	112	91	(2.2–10)		11	101	(0.7–5.5)	
Metaplasia	≥ 1	17	0	—	0.0004	3	14	1.5	N.S.
	0	156	102			19	137	(0.3–6.3)	
Antrum and corpus									
OLGA stages	≥ I	128	42	4.0	< 0.0001	20	108	4.0	N.S.
	0	45	60	(2.3–7.1)		2	43	(0.9–36)	
OLGIM stages	≥ I	62	4	13.6	< 0.0001	14	48	3.7	0.0075
	0	111	98	(4.8–53)		8	103	(1.4–11)	

Abbreviations: 95% CI, 95% confidence interval; Metaplasia, intestinal metaplasia; N.S., not significant; OLGA, Operative Link on Gastritis Assessment; OLGIM, Operative Link on Gastric Intestinal Metaplasia Assessment.

* Fisher's exact test.

Additional analyzes comparing East Asian‐ and Western‐type infections in Hp‐infected Japanese Brazilians revealed no statistically significant differences in OLGA/OLGIM stages, with ORs (95% CI) of 4.2 (0.4–62.5) and 2.3 (0.4–15.6), respectively (*n* = 29). Similarly, comparisons between Japanese and non‐Japanese Brazilians with Western‐type infections showed no significant differences in OLGA/OLGIM stages, with ORs (95% CI) of 0.8 (0.2–5.1) and 1.8 (0.3–8.7), respectively (*n* = 151). Although the total number of Hp‐infected cases was small among Japanese Brazilians, these results showed that within this cohort, CagA type and ethnicity alone were not major determinants of gastric pathology severity (Table S4).

### 3.4 Hp Classification and Serum Markers

**FIGURE 2:**
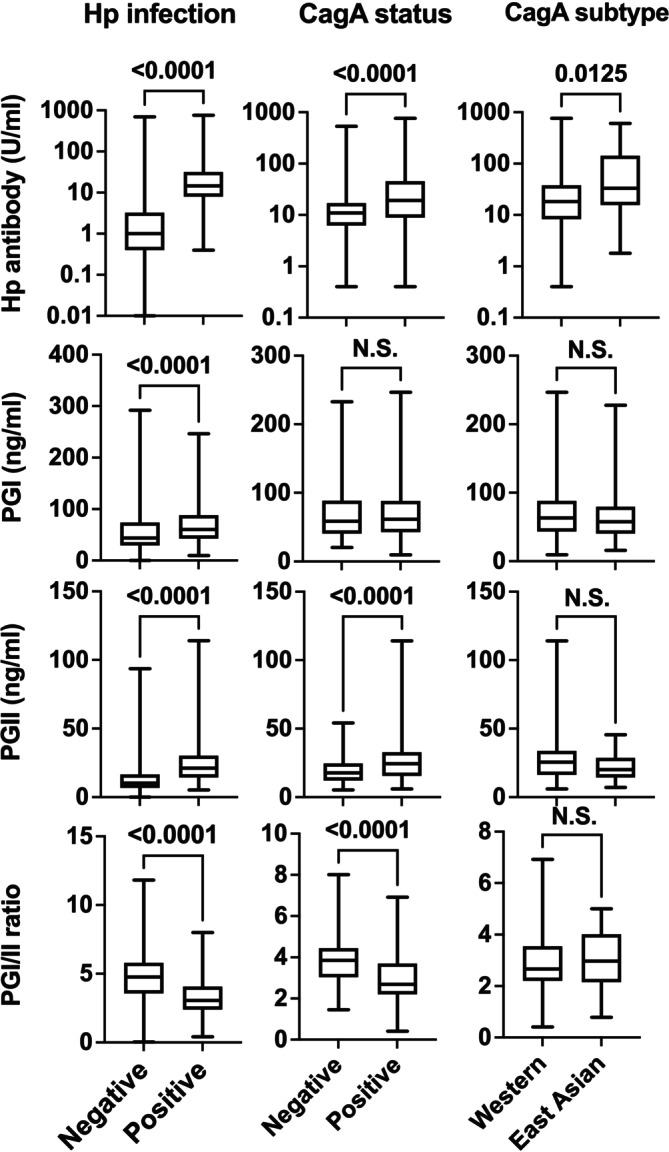
Serum marker levels and ratios in 577 patients with successful immunohistochemical classification of *Helicobacter pylori* (Hp). Serum levels of Hp antibody, pepsinogen I (PGI), and pepsinogen II (PGII), and the PGI/II ratio were compared among groups defined by Hp infection status (positive or negative), CagA status (positive or negative), and CagA subtypes (East Asian type or Western type). *p* values were calculated using the Mann–Whitney *U* test. N.S. indicates not significant (*p* ≥ 0.05).

To examine the effect of CagA status and subtypes on serum markers, we compared their distribution across groups defined by IHC‐based Hp classification (Figure 2). Serum Hp antibody, PGI, and PGII levels were significantly higher, while PGI/II ratios were significantly lower, in Hp‐infected patients than in noninfected patients (*p* < 0.0001 for all comparisons). Patients with CagA(+) infection showed significantly higher Hp antibody and PGII levels, and lower PGI/II ratios than those with CagA(−) infection (*p* < 0.0001 for all comparisons), although PGI levels did not differ between the two groups.

Patients with East Asian‐type infection exhibited significantly higher Hp antibody levels than those with Western‐type infection (*p* = 0.0125), while PGI and PGII levels, and PGI/II ratios did not differ significantly between the two groups. Similar trends were observed in the subset of 472 patients with concordant IHC and PCR classification (Figure S1).

### 3.5 Serum Markers and Gastric Pathology

Gastric pathology scores (Sydney System) and stages (OLGA/OLGIM) were compared with serum markers (Hp antibody, PGI, PGII, and PGI/II ratio) in 586 biopsy samples (Table S5). The relationship between these serum markers and gastric pathology was also analyzed according to the ABC classification (Table 3). Groups B and C/D showed significantly higher mean activity/inflammation scores in both the antrum and corpus compared to Group A. Groups C/D also had significantly higher mean OLGA/OLGIM stages and higher mean corpus inflammation scores than Groups A or B. Group B showed significantly higher mean antral and corpus activity/inflammation scores, as well as higher mean antral atrophy scores and OLGA stages, compared to Group A.

**TABLE 3:** Comparison of gastric pathology scores and stages among ABC classification groups in a cohort of 586 patients.

	ABC classification groups in the 586 patients						
	Group A	Group B	Groups C/D	Kruskal–Wallis test, *p* value	Dunn's multiple comparison test, *p* value		
	*n* = 246	*n* = 198	*n* = 142		Group A vs. B	Group(s) A vs. C/D	Group(s) B vs. C/D
Descriptive statistics, mean (median)							
Antrum scores							
Activity	0.2 (0)	1.1 (1)	1.1 (1)	< 0.0001	< 0.0001	< 0.0001	N.S.
Inflammation	0.5 (0)	1.4 (1)	1.4 (1)	< 0.0001	< 0.0001	< 0.0001	N.S.
Atrophy	0.2 (0)	0.6 (0)	0.6 (0)	< 0.0001	< 0.0001	< 0.0001	N.S.
Metaplasia	0.2 (0)	0.3 (0)	0.4 (0)	0.0001	N.S.	< 0.0001	0.0086
Corpus scores							
Activity	0.1 (0)	0.8 (1)	1.0 (1)	< 0.0001	< 0.0001	< 0.0001	N.S.
Inflammation	0.3 (0)	1.0 (1)	1.2 (1)	< 0.0001	< 0.0001	< 0.0001	0.0348
Atrophy	0.1 (0)	0.2 (0)	0.6 (0)	< 0.0001	N.S.	< 0.0001	< 0.0001
Metaplasia	0.0 (0)	0.0 (0)	0.3 (0)	< 0.0001	N.S.	< 0.0001	< 0.0001
Antrum and corpus							
OLGA stages	0.3 (0)	0.6 (0)	0.9 (1)	< 0.0001	< 0.0001	< 0.0001	0.0003
OLGIM stages	0.2 (0)	0.3 (0)	0.6 (0)	< 0.0001	N.S.	< 0.0001	< 0.0001

Abbreviations: Groups C/D, combined Groups C and D for the analysis; Metaplasia, intestinal metaplasia; N.S., not significant; OLGA, Operative Link on Gastritis Assessment; OLGIM, Operative Link on Gastric Intestinal Metaplasia Assessment.

To evaluate the diagnostic performance of the ABC method, we calculated the sensitivity, specificity, positive predictive value (PPV), and negative predictive value (NPV) (Table 4). The pepsinogen test alone (Groups C/D) showed relatively low sensitivities for OLGA/OLGIM stages ≥ I (37%/44%), ≥ II (54%/56%), and ≥ III (67%/40%). In contrast, Groups B/C/D—positive for the pepsinogen and/or Hp antibody tests—showed higher sensitivities of 76%/73%, 87%/78%, and 83%/73%, and NPVs of 76%/86%, 97%/97%, and 100%/98%, respectively. For detecting antrum/corpus inflammation scores ≥ 1, ≥ 2, and 3, Groups B/C/D showed sensitivities of 75%/79%, 91%/94%, and 95%/100%, and NPVs of 59%/70%, 94%/98%, and 99%/100%, respectively.

**TABLE 4:** Sensitivities and negative predictive values for OLGA/OLGIM stages and antral/corpus inflammation scores across ABC classification groups in a cohort of 586 patients.

ABC test used for classification	Pepsinogen test alone				Pepsinogen and/or Hp antibody tests			
Positive or negative for the test	Positive	Negative	Positive	Negative	Positive	Negative	Positive	Negative
ABC classification group(s)	C/D	A/B	C/D	A/B	B/C/D	A	B/C/D	A
For OLGA/OLGIM stages	OLGA		OLGIM		OLGIM		OLGA	
For detecting stages ≥ I								
Stages I, II, III, and IV (*n*)	94	157	56	72	192	59	94	34
Stage 0 (*n*)	48	287	86	372	148	187	246	212
*p* *	< 0.0001		< 0.0001		< 0.0001		0.0001	
Sensitivity (%)	37		44		76		73	
Specificity (%)	86		81		56		46	
PPV (%)	66		39		56		28	
NPV (%)	65		84		76		86	
For detecting stages ≥ II								
Stages II, III, and IV (*n*)	34	29	18	14	55	8	25	7
Stage 0 and I (*n*)	108	415	124	430	285	238	315	239
*p* *	< 0.0001		0.0001		< 0.0001		0.0256	
Sensitivity (%)	54		56		87		78	
Specificity (%)	79		78		46		43	
PPV (%)	24		13		16		7	
NPV (%)	93		97		97		97	
For detecting stages ≥ III								
Stages III and IV (*n*)	4	2	6	9	5	1	11	4
Stage 0, I, and II (*n*)	138	442	136	435	335	245	329	242
*p* *	0.0329		N.S.		N.S.		N.S.	
Sensitivity (%)	67		40		83		73	
Specificity (%)	76		76		42		42	
PPV (%)	3		4		1		3	
NPV (%)	100		98		100		98	
For inflammation scores in the	Antrum		Corpus		Antrum		Corpus	
For detecting scores ≥ 1								
Scores 1, 2, 3, and 4 (*n*)	120	285	124	237	305	100	286	75
Score 0 (*n*)	22	159	18	207	35	146	54	171
*p* *	< 0.0001		< 0.0001		< 0.0001		< 0.0001	
Sensitivity (%)	30		34		75		79	
Specificity (%)	88		92		81		76	
PPV (%)	85		87		90		84	
NPV (%)	36		47		59		70	
For detecting scores ≥ 2								
Scores 2 and 3 (*n*)	63	96	43	38	144	15	76	5
Scores 0 and 1 (*n*)	79	348	99	406	196	231	264	241
*p* *	< 0.0001		< 0.0001		< 0.0001		< 0.0001	
Sensitivity (%)	40		53		91		94	
Specificity (%)	81		80		54		48	
PPV (%)	44		30		42		22	
NPV (%)	78		91		94		98	
For detecting score 3								
Score 3 (*n*)	22	20	8	6	40	2	14	0
Scores 0, 1, and 2 (*n*)	120	424	134	438	300	244	326	246
*p* *	< 0.0001		0.0077		< 0.0001		0.0005	
Sensitivity (%)	52		57		95		100	
Specificity (%)	78		77		45		43	
PPV (%)	15		6		12		4	
NPV (%)	95		99		99		100	

*Note:* Groups C/D, A/B, and B/C/D refer to combined Groups C and D; A and B; and B, C, and D (i.e., groups other than Group A), respectively, for the analysis.

Abbreviations: N.S., not significant; NPV, negative predictive value; OLGA, Operative Link on Gastritis Assessment; OLGIM, Operative Link on Gastric Intestinal Metaplasia Assessment; PPV, positive predictive value.

* Fisher's exact test.

To assess potential ethnic differences in ABC method performance, we compared diagnostic parameters between Japanese and non‐Japanese Brazilians (Table S6). The prevalence of Hp infection was lower in Japanese Brazilians (38/122, 31%) than in non‐Japanese Brazilians (237/455, 52%). The sensitivity for detecting OLGA/OLGIM ≥ I was lower in Japanese Brazilians (61%/62%) compared to non‐Japanese Brazilians (81%/79%), while specificity and PPV were higher. Notably, among Japanese Brazilians, Group A (ABC test–negative) included more cases with OLGA/OLGIM ≥ II, resulting in lower NPV (93%/93%) compared to non‐Japanese Brazilians (98%/99%).

### 3.6 Serum Markers, Gastric Pathology, and Hp Classification

ROC analyzes of serum markers for gastric pathology were conducted according to CagA status and subtypes—CagA(−), Western‐type, East Asian‐type, and total Hp‐positive cases—based on IHC‐based Hp classification (Figure S2). Optimal cutoffs for serum markers differed between CagA(+) and CagA(−) infections, as well as between East Asian‐ and Western‐type infections. Across all cases, regardless of CagA status and subtypes, the optimal cutoff values were as follows: Hp antibody, 4.2 (Hp infection); PGI/II ratio, 3.0 (OLGA) or 2.7 (OLGIM); PGI, 98 (OLGA) or 35 (OLGIM); and PGII, 19 (corpus activity) or 17 (corpus inflammation).

To examine population‐specific variation, ROC analyzes were also performed separately for Japanese and non‐Japanese Brazilians (Figure S3). The optimal Hp antibody cutoff for infection was 8.0 (AUC: 0.93) in Japanese Brazilians and 4.2 (AUC: 0.88) in non‐Japanese Brazilians. For OLGA/OLGIM staging, the PGI and PGI/II ratio cutoffs were 75.9/34.7 and 5.2/4.9, respectively, in Japanese Brazilians, and 41.2/30.2 and 3.0/2.6, respectively, in non‐Japanese Brazilians.

## 4 Discussion

Using a novel immunohistochemical method, we achieved a 98% success rate in classifying Hp infection status, CagA expression, and CagA subtypes in FFPE gastric biopsy samples from Brazil, with 577 of 586 samples yielding interpretable results. This high success rate underscores the feasibility of IHC‐based classification of CagA status and subtypes for both large‐scale prospective and retrospective studies. In this endoscopy‐referred cohort, the prevalence of Hp infection was 48%, with Western‐type CagA(+) (26%) and CagA(−) (18%) strains being most prevalent. East Asian‐type CagA(+) infection (4%) occurred predominantly in Japanese Brazilians. The inclusion of Japanese Brazilians, who comprised 21% of our cohort, enabled the evaluation of Hp strain diversity within an ethnically distinct subgroup. Although this proportion exceeds their national representation (1.1%), it reflects the patient demographics of the participating hospitals in Brazil and allowed the investigation of East Asian‐type infections in a non‐Asian setting.

Patients with CagA(+) Hp infection exhibited a distinct clinicopathologic profile characterized by more frequent corpus activity/inflammation, higher OLGA/OLGIM stages, higher Hp antibody and PGII levels, and lower PGI/II ratios. East Asian‐type CagA(+) infections were particularly associated with increased antral intestinal metaplasia, higher OLGIM stages, and elevated Hp antibody levels. These findings underscore the distinct pathological and serological features associated with CagA positivity and East Asian‐type strains. They highlight the clinical relevance of considering CagA status and subtypes in refining risk stratification methods for gastric cancer screening and prevention.

Notably, the ABC method demonstrated high sensitivity and negative predictive value for gastric pathology, even in a cohort with diverse Hp strains, including a substantial proportion of CagA(−) and Western‐type infections. Although originally developed in Japan—where nearly all strains are East Asian‐type CagA(+) [21]—the method proved effective in this more heterogeneous setting, supporting its broader applicability to regions such as Europe and Latin America [22, 23]. CagA‐negative infections, which account for up to 40% of cases in Western countries, have raised concerns regarding the generalizability of CagA‐based risk models [24]. Our findings provide empirical evidence that the ABC method retains clinical value even in such diverse populations, reinforcing its potential role in global gastric cancer risk stratification strategies.

This study provides the first documentation of East Asian‐type Hp infection among Japanese Brazilians, with notable clustering in second‐generation individuals. This pattern suggests possible intrafamilial transmission; however, the absence of bacterial genome sequencing or direct transmission tracking limits definitive conclusions. Alternative explanations, including shared environmental exposure, dietary habits, and social interactions, may also contribute to this clustering. We observed a generational decline in East Asian‐type infections, indicating a gradual replacement by Western‐type strains. The clustering observed in second‐generation individuals may reflect a combination of intrafamilial transmission and evolving infection dynamics over generations. Although Hp is primarily transmitted through person‐to‐person contact, other transmission routes are documented [25]. Maternal transmission has been proposed as a dominant pathway [26], however, our findings do not support a maternal bias, as more second half‐generation individuals with East Asian‐type infection had Japanese fathers than mothers.

Hp CagA(−) infections were more prevalent in the broader Brazilian population, beyond East Asian‐type infections, which were largely confined to Japanese and other East Asian Brazilians. This predominance of CagA(−) and Western‐type infections reflects greater strain diversity in Brazil and contrasts with Japan, where nearly all strains are CagA(+) [27, 28]. In our cohort, 62% of Hp‐infected individuals in São Paulo and 64% in Brasília harbored CagA(+) strains. Among these, 13% were East Asian‐type, mostly found in Japanese Brazilians. In contrast, a study from the Brazilian Amazon Region reported a higher CagA positivity rate of 80%, with nearly all CagA(+) strains classified as Western‐type based on EPIYA motif analysis [12]. These findings underscore substantial geographic variability in both the prevalence and composition of CagA(+) Hp strains across Brazil.

CagA, a major Hp virulence factor [29, 30], promotes gastric mucosal injury by disrupting intracellular signaling through tyrosine phosphorylation and SHP‐2 activation [31, 32]. CagA(+) strains are known to induce more severe gastric pathology than CagA(−) strains [33]. In our study, consistent with a previous Brazilian report using fresh biopsy samples [12], CagA(+) infection was associated with increased corpus activity/inflammation, which are precursors for diffuse‐type gastric cancer [14], as well as elevated OLGA/OLGIM stages, which are linked with intestinal‐type gastric cancer [15]. These findings reinforce the pathogenic role of CagA in gastric carcinogenesis by contributing to both inflammatory and atrophic processes and provide a mechanistic rationale for incorporating CagA status into risk stratification models.

This study is the first to report differences in Hp CagA subgroups within a single population in Brazil. Similar subtype diversity has been observed in Thailand [34, 35, 36] and Okinawa, Japan [37, 38, 39]. In Okinawa, all isolates from patients with gastric cancer or duodenal ulcer were CagA(+), and East Asian‐type strains were significantly more prevalent in gastric cancer patients than in those with duodenal ulcers [33]. CagA(+) strains with EPIYA‐D motifs (East Asian‐type) are thought to contribute to the high incidence of gastric cancer in East Asian countries, while those with multiple EPIYA‐C motifs (Western‐type) are associated with atrophic gastritis and increased cancer risk [40, 41]. Although IHC cannot determine the number of EPIYA‐C repeats, it reliably distinguishes East Asian‐ and Western‐type strains based on motif detection. In our study, East Asian‐type CagA infection was more frequently associated with antral intestinal metaplasia and higher OLGIM stages, likely due to its greater oncogenic potential, particularly enhanced SHP2 activation and inflammatory responses [21, 42].

However, when analyzed separately in Japanese and non‐Japanese Brazilians, these differences were not statistically significant. Among Japanese Brazilians infected with East Asian‐type strains, the ORs for OLGA and OLGIM stages were elevated (4.2 and 2.3, respectively), indicating a possible trend despite the lack of statistical significance, likely due to limited sample size (*n* = 29). In contrast, Western‐type infections exhibited little ethnic variation (*n* = 151), with ORs approximating unity. These findings suggest that CagA subtype may influence gastric pathology to some extent, whereas the effect of ethnicity appears limited, especially in cases involving Western‐type strains. Furthermore, additional factors, including host genetics [43], immune response [44], and environmental exposures [45], are also likely to contribute to gastric pathology progression.

The recently validated immunohistochemical method [16] demonstrated high reliability, achieving a 98% success rate and 82% concordance with PCR. Given that formalin fixation often leads to DNA fragmentation and cross‐linking [46], which reduces PCR sensitivity [47], IHC presents a practical alternative for identifying CagA status and subtypes in FFPE tissues. Additionally, the risk of contamination from prevalent CagA strains in FFPE samples during routine tissue processing may compromise PCR accuracy [48], whereas IHC allows direct visualization of bacterial localization on the surface of gastric foveolar epithelium, minimizing misclassification. Despite its advantages, IHC‐based Hp classification requires further validation across different settings to confirm its reproducibility. Variability in sample processing protocols and inter‐laboratory differences may affect diagnostic consistency. Standardization of staining protocols and inter‐laboratory comparison studies will be crucial for ensuring reliability in broader applications.

Among the 586 samples examined, IHC‐based CagA typing failed in nine cases (1.5%). Specifically, six cases were both Hp(+) and CagA(+) but EPIYA‐C(−)/EPIYA‐D(−), suggesting the presence of an alternative CagA variant, potentially the EPIYA‐AB type [12]. Additionally, three cases were Hp(+), EPIYA‐C(+), and EPIYA‐D(−), but CagA(−), likely due to the limited sensitivity of the commercially available anti‐CagA mAb used [16]. Nevertheless, analyzes of the 577 successfully classified cases by IHC and the 472 cases with concordant IHC and PCR classification revealed consistent associations between Hp strain types, gastric pathology, and serum markers. These results further underscore the robustness of our immunohistochemical approach while also leaving areas for methodologic refinement in future studies, including the development of more sensitive anti‐CagA mAb for FFPE specimens.

Serum pepsinogens are widely utilized as noninvasive biomarkers for detecting gastric mucosal atrophy. PGI is primarily secreted by gastric chief cells and mucous neck cells in the fundic gland, whereas PGII is secreted not only by fundic glands but also by pyloric glands of the antrum and duodenal mucosa [49, 50]. During Hp infection, progressive mucosal atrophy leads to decreased PGI secretion, while PGII production remains relatively unchanged or increases, ultimately resulting in a reduced PGI/II ratio [51]. This pattern is particularly pronounced in advanced atrophic gastritis. In our study, patients with CagA(+) infections exhibited higher Hp antibody and PGII levels and lower PGI/II ratios compared to those with CagA(−) infections. These findings are consistent with more frequent corpus activity/inflammation and higher OLGA/OLGIM stages. However, differences in antral intestinal metaplasia and OLGIM stages between East Asian and Western‐type infections were not reflected in the serum PGI/II ratios. This finding suggests that serum pepsinogens may not adequately capture subtype‐specific intestinal metaplasia. This limitation may partly explain the higher optimal PGI/II cutoff observed in Japanese Brazilians. A more detailed analysis of this cutoff issue and its impact on risk stratification is discussed later in this section.

The ABC classification, using manufacturer‐recommended cutoff values, effectively stratified gastric cancer risk in our cohort, with significant differences observed in OLGA/OLGIM stages and activity/inflammation scores across risk groups. These findings demonstrate its clinical utility in the Brazilian population. Recent European guidelines emphasize the use of combined serologic markers for risk stratification and endorse biomarker‐based approaches as viable alternatives to mass endoscopic screening [9]. In parallel, global perspectives highlight the well‐established role of the ABC method in Japan while underscoring the need for further validation in diverse populations with mixed Hp genotypes [10]. Our findings contribute to this ongoing evaluation by demonstrating the method's applicability in a Brazilian cohort with diverse Hp infections, while also recognizing the necessity of population‐specific adaptations for optimizing its effectiveness.

To compare patients with different CagA status and subtypes, we defined unfavorable histology as Sydney System grading scores ≥ 1 or OLGA/OLGIM stages ≥ I. This threshold was selected based on the average distribution of pathology scores and stages, and the low frequency of patients classified as high‐risk [52] (OLGA/OLGIM stages III and IV), which accounted for only 1.0% and 2.5% of the total, respectively, in our Brazilian cohort. However, two recent Brazilian studies have questioned the reliability of serum pepsinogen testing for distinguishing high‐ from low‐risk patients, reporting low sensitivity for detecting gastric atrophy when using OLGA staging [53, 54].

Consistent with these findings, our study also showed that the sensitivity of the pepsinogen test alone for detecting OLGA/OLGIM stages ≥ I to ≥ III was relatively low, ranging from 37% to 67%. In contrast, the ABC method demonstrated substantially higher sensitivity (73%–87%) in groups other than Group A (negative for both tests). It also showed consistently high negative predictive values (NPVs): 97%/97% or 98%/100% for detecting OLGA/OLGIM stages ≥ II or ≥ III, respectively. Similarly, NPVs were 94%/98% or 99%/100% for detecting antrum or corpus inflammation scores ≥ 2 or 3, respectively. These findings further underscore the utility of the ABC method for reliably identifying low risk individuals (Group A), regardless of CagA status and subtypes. They also support its potential role in reducing unnecessary endoscopic examinations in screening programs, thereby improving screening efficiency not only in Brazil but potentially in other regions with diverse Hp strain distributions.

Although optimal cutoff values for serum markers varied by CagA status, subtype, and possibly host‐related factors, the values identified across all cases generally aligned with manufacturer‐recommended thresholds, which appeared broadly applicable in the Brazilian population. However, some OLGA/OLGIM ≥ II cases were observed even among Group A individuals (ABC test–negative), resulting in lower NPVs among Japanese Brazilians (93% for OLGA and 93% for OLGIM) compared to non‐Japanese Brazilians (98% for OLGA and 99% for OLGIM). This discrepancy was primarily due to differences in the optimal PGI/II ratio cutoff. ROC analysis indicated that the optimal cutoff for detecting OLGA/OLGIM was higher in Japanese Brazilians (5.2/4.9) than in non‐Japanese Brazilians (3.0/2.6). Applying a uniform cutoff of 3.0 therefore led to underestimation of gastric mucosal atrophy and intestinal metaplasia in the Japanese Brazilian group. This “false Group A” phenomenon, in which individuals classified as ABC test‐negative still exhibit significant gastric mucosal atrophy and intestinal metaplasia, has been previously reported in relatively ethnically homogeneous populations, such as Japan [55] and Korea [56]. Our findings reinforce that this phenomenon can also affect multiethnic populations, highlighting the need to optimize cutoff values based on population‐specific biomarker distributions to improve gastric cancer risk stratification globally.

This study has several limitations. First, it was conducted in an endoscopy‐referred cohort from selected regions of Brazil, including a subset of asymptomatic individuals (12%), which may limit the generalizability of the findings. Second, the small number of East Asian‐type Hp infections (*n* = 22) reduced the statistical power for subgroup analyzes. Third, the cross‐sectional design precluded assessment of disease progression and long‐term gastric cancer risk. Fourth, the absence of Hp‐negative individuals and matched family members limited the evaluation of intrafamilial transmission and precluded analysis of maternal predominance. Fifth, although the participation rate was high, non‐responders were not formally assessed, and the possibility of selection bias cannot be excluded. Finally, potential confounding factors—such as co‐infections (e.g., Epstein–Barr virus), lifestyle factors (e.g., smoking, diet, and alcohol), and host genetic background—were not controlled for and may have influenced gastric pathology and biomarker variability. Future studies incorporating longitudinal follow‐up, detailed lifestyle profiling, and multi‐center comparisons across diverse populations will be essential to clarify the independent impact of CagA status and subtypes, and to validate the clinical utility of the ABC method in broader gastric cancer screening programs.

## 5 Conclusions

Our findings underscore the clinical utility of the ABC method as a noninvasive strategy for identifying individuals at low risk of gastric cancer, even in populations with diverse Hp CagA status and subtypes. Despite its initial development in Japan—where East Asian‐type CagA(+) strains predominate—the method showed robust performance in our Brazilian cohort, which included a high proportion of CagA(−) and Western‐type infections.

Immunohistochemical classification of CagA status and subtypes revealed that CagA(+) infections were associated with more advanced gastric pathology than CagA(−) infections, including increased corpus activity/inflammation and higher OLGA/OLGIM stages, accompanied by lower PGI/II ratios. Among CagA(+) strains, East Asian‐type was linked to greater histologic severity, particularly increased antral intestinal metaplasia and higher OLGIM stages. However, these subtype‐specific changes were not consistently reflected in the PGI/II ratio, indicating a limitation of serologic biomarkers in capturing subtype‐specific pathology.

ABC classification groups showed significant differences in OLGA/OLGIM stages and activity/inflammation scores, supporting its utility in stratifying gastric cancer risk based on histologic findings. The ABC method demonstrated high negative predictive values (up to 100%) for excluding clinically significant gastric lesions. However, a subset of test‐negative individuals (Group A), especially among Japanese Brazilians, exhibited OLGA/OLGIM stage ≥ II. This “false Group A” pattern, consistent with findings from Japan and Korea, was linked to a higher optimal PGI/II cutoff in this subgroup, emphasizing the need for population‐specific adaptation of serologic thresholds.

Because of its high reliability and compatibility with FFPE tissue, immunohistochemistry is a practical alternative to molecular methods for CagA classification, especially when such methods are not feasible. Beyond its current use, this method could support future international studies exploring associations among Hp infection status, CagA status, CagA subtype, gastric pathology, and serum markers. Its feasibility across diverse clinical settings may enable standardized cross‐population comparisons and expand biomarker‐based risk stratification in gastric cancer prevention.

With appropriate adjustment of cutoff values, the ABC method holds promise as a risk stratification tool for use in international gastric cancer screening programs. Therefore, future studies incorporating longitudinal follow‐up, detailed lifestyle profiling, and multi‐center comparisons across diverse populations will be essential to validate the generalizability of the ABC method and to optimize its implementation in broader screening frameworks.

## Author Contributions

**Luis Masuo Maruta:** conceptualization (equal), data curation (equal), formal analysis (equal), investigation (equal), methodology (equal), project administration (equal), resources (equal), supervision (equal), validation (equal), writing – original draft (equal). **Asuka Furukawa:** conceptualization (equal), data curation (equal), formal analysis (equal), investigation (equal), methodology (equal), supervision (equal), validation (equal), visualization (equal), writing – original draft (equal). **Heinrich Bender Kohnert Seidler:** project administration (equal), resources (equal). **Aloisio Felipe‐Silva:** data curation (equal), resources (equal). **Keisuke Uchida:** data curation (equal), formal analysis (equal), investigation (equal), methodology (equal), validation (equal), writing – review and editing (supporting). **Daisuke Kobayashi:** data curation (equal), formal analysis (equal), investigation (equal), methodology (equal), validation (equal), writing – review and editing (supporting). **Kurara Yamamoto:** investigation (supporting), writing – review and editing (supporting). **Junko Minami:** investigation (supporting), methodology (supporting), writing – review and editing (supporting). **Masaki Sekine:** investigation (supporting). **Minako Takagi:** investigation (supporting), methodology (supporting). **Renato Takayuki Hassegawa:** investigation (supporting), resources (equal). **Eduardo Koji Marchi Ogawa:** investigation (supporting), resources (equal). **Rodrigo Barbosa Villaça:** investigation (supporting), resources (equal). **Tecio de Araujo Couto:** investigation (supporting), resources (equal). **Jorge Alberto Capra Biasuz:** investigation (supporting), resources (equal). **Edmar Tafner:** investigation (supporting), resources (equal). **Ana Luiza Werneck‐Silva:** investigation (supporting), resources (equal). **Simone Perez Pilli:** investigation (supporting), resources (equal). **José Guilherme Nogueira da Silva:** investigation (supporting), resources (equal). **Leonard Medeiros da Silva:** formal analysis (supporting), resources (supporting). **Ricardo Ambrosio Fock:** investigation (supporting), resources (supporting), writing – review and editing (supporting). **Chinatsu Ogura:** investigation (supporting), methodology (supporting). **Yumi Mizuguchi:** investigation (supporting), methodology (supporting). **Keiko Miura:** investigation (supporting), writing – review and editing (supporting). **Kouhei Yamamoto:** writing – review and editing (supporting). **Yoshinobu Eishi:** conceptualization (equal), formal analysis (equal), investigation (equal), methodology (equal), project administration (equal), supervision (equal), validation (equal), writing – original draft (equal). **Kenichi Ohashi:** conceptualization (equal), funding acquisition (equal), project administration (equal), supervision (equal), validation (equal), writing – review and editing (equal).

## Ethics Statement

This study was approved by the Ethics Committee of Hospital Universitário da Universidade de São Paulo (HUUSP CEP76, CAAE 52744016.7.0000.0076) and the Ethics Committee of Institute of Science Tokyo Hospital (formerly known as Tokyo Medical and Dental University Hospital; M2018‐244 and M2019‐072). Written informed consent was obtained from all participants prior to enrollment. The study was conducted in accordance with the Declaration of Helsinki and relevant national guidelines for research involving human participants.

## Conflicts of Interest

Kenichi Ohashi has received research funding over the last 4 years, and Yoshinobu Eishi has received consulting fees over the last 5 years from Eiken Chemical Corporation, Tokyo, Japan.

## Supporting information


**Figure S1.** Serum marker levels and ratios in 472 patients with concordant *Helicobacter pylori* (Hp) classification by immunohistochemistry and polymerase chain reaction.

**Figure S2.** Receiver operating characteristic (ROC) curves of serum markers for gastric pathology across subgroups defined by immunohistochemical CagA typing.

**Figure S3.** Receiver operating characteristic (ROC) curves of serum markers for gastric pathology in Japanese Brazilians and non‐Japanese Brazilians.

**Table S1.** Clinicopathologic characteristics of all patients and patient subgroups included in the study.

**Table S2.** Concordance between *Helicobacter pylori* classification by immunohistochemistry and polymerase chain reaction in 577 samples with successful immunohistochemical classification.

**Table S3.** Comparison of gastric pathology scores and stages among 472 patients categorized by CagA status and subtype, based on concordant classification by immunohistochemistry and polymerase chain reaction.

**Table S4.** Comparison of gastric pathology (i) between East Asian‐type and Western‐type infections in Japanese Brazilians, and (ii) between Japanese and non‐Japanese Brazilians with Western‐type infection.

**Table S5.** Correlation between serum markers and gastric pathology scores and stages in the 586 patients included in the study.

**Table S6.** Performance of the ABC method for detecting OLGA and OLGIM stages in Japanese and non‐Japanese Brazilians.

## Data Availability

The data used and/or analyzed during the study can be obtained from the corresponding author on reasonable request.
