# Supplementary material for: Validation of the ABC Method for Gastric Cancer Risk Stratification Across Helicobacter pylori Infections With Diverse CagA Status and Subtypes in Brazil
Source: Cancer Med. 2025 Jun 27;14(13):e71016. doi: 10.1002/cam4.71016 (PMC12203232; doi:10.1002/cam4.71016)
Supplement: Supplementary file 1 — Figure S1. Serum marker levels and ratios in 472 patients with concordant Helicobacter pylori (Hp) classification by immunohistochemistry and polymerase chain reaction. [file CAM4-14-e71016-s004.docx]

**Supplementary Figure S1**: Serum marker levels and ratios in 472 patients with concordant *Helicobacter pylori* (Hp) classification by immunohistochemistry and polymerase chain reaction. Serum levels of Hp antibody, pepsinogen I (PGI), pepsinogen II (PGII), and the PGI/II ratio were compared among groups defined by Hp infection status (positive or negative), CagA status (positive or negative), and CagA-positive subtypes (East Asian type or Western type). P values were calculated using the Mann–Whitney U test. N.S., not significant (P ≥ 0.05).
