# Supplementary material for: Validation of the ABC Method for Gastric Cancer Risk Stratification Across Helicobacter pylori Infections With Diverse CagA Status and Subtypes in Brazil
Source: Cancer Med. 2025 Jun 27;14(13):e71016. doi: 10.1002/cam4.71016 (PMC12203232; doi:10.1002/cam4.71016)
Supplement: Supplementary file 2 — Figure S2. Receiver operating characteristic (ROC) curves of serum markers for gastric pathology across subgroups defined by immunohistochemical CagA typing. [file CAM4-14-e71016-s009.docx]

**Supplementary Figure S2**: Receiver operating characteristic (ROC) curves of serum markers for gastric pathology across subgroups defined by immunohistochemical CagA typing. The serum markers analyzed included *Helicobacter pylori* (Hp) antibody, pepsinogen I (PGI), pepsinogen II (PGII), and the PGI/II ratio. These were evaluated in relation to various gastric pathology parameters (Hp infection; OLGA and OLGIM stages; and activity or inflammation in the antrum and corpus) across subgroups defined as CagA-negative, Western-type, East Asian-type, and all Hp-infected patients. The area under the curve (AUC) was calculated from the ROC curves, and optimal cutoff values were determined using the Youden Index.
