# Supplementary material for: Validation of the ABC Method for Gastric Cancer Risk Stratification Across Helicobacter pylori Infections With Diverse CagA Status and Subtypes in Brazil
Source: Cancer Med. 2025 Jun 27;14(13):e71016. doi: 10.1002/cam4.71016 (PMC12203232; doi:10.1002/cam4.71016)
Supplement: Supplementary file 3 — Figure S3. Receiver operating characteristic (ROC) curves of serum markers for gastric pathology in Japanese Brazilians and non‐Japanese Brazilians. [file CAM4-14-e71016-s002.docx]

**Supplementary Figure S3**: Receiver operating characteristic (ROC) curves of serum markers for gastric pathology in Japanese Brazilians and non-Japanese Brazilians. The serum markers analyzed included *Helicobacter pylori* (Hp) antibody, pepsinogen I (PGI), and the PGI/II ratio. Associations with gastric pathology parameters (Hp infection, OLGA stage, and OLGIM stage) were evaluated separately in Japanese and non-Japanese Brazilians. The area under the curve (AUC) was calculated from ROC curves, and optimal cutoff values were determined using the Youden Index.
