# Supplementary material for: Validation of the ABC Method for Gastric Cancer Risk Stratification Across Helicobacter pylori Infections With Diverse CagA Status and Subtypes in Brazil
Source: Cancer Med. 2025 Jun 27;14(13):e71016. doi: 10.1002/cam4.71016 (PMC12203232; doi:10.1002/cam4.71016)
Supplement: Supplementary file 4 — Table S1. Clinicopathologic characteristics of all patients and patient subgroups included in the study. [file CAM4-14-e71016-s003.docx]

**Supplementary Table S1:** Clinicopathologic characteristics of all patients and patient subgroups included in the study.

|  |  | | Patients examined by IHC and PCR | Patients with successful classification by IHC* | Patients with concordant classification by IHC and PCR* |
| --- | --- | --- | --- | --- | --- |
| Number |  | | 586 | 577 | 472 |
| Age, year (mean ± SD) | | | 55.0 ± 13.8 | 55.0 ± 13.8 | 55.1 ± 13.9 |
| Subject (number, Male / Female) | | | 253 / 333 | 250 / 327 | 199 / 273 |
| Ethnicity (Japanese Brazilians / Non-Japanese Brazilians) | | | 125 / 461 | 122 / 455 | 107 / 365 |
| Symptoms (number) |  | |  |  |  |
| Dyspepsia or epigastric pain | | | 271 | 265 | 219 |
| Asymptomatic |  | | 69 | 68 | 62 |
| Gastroesophageal reflux | |  | 25 | 25 | 22 |
| Abdominal pain |  | | 7 | 7 | 3 |
| Others |  | | 17 | 17 | 15 |
| Unknown |  | | 197 | 195 | 151 |
| Endoscopic atrophy (number) | | |  |  | 472 |
| Kimura-Takemoto | C-0 | | 170 | 168 | 135 |
|  | C-1 | | 87 | 87 | 76 |
|  | C-2 | | 55 | 52 | 46 |
|  | C-3 | | 31 | 31 | 26 |
|  | O-1 | | 14 | 14 | 12 |
|  | O-2 | | 14 | 14 | 11 |
|  | O-3 | | 19 | 17 | 15 |
| Unknown |  | | 196 | 194 | 151 |
| Histologic atrophy (number) | | |  |  |  |
| OLGA | Stage 0 | | 335 | 331 | 284 |
|  | Stage I | | 188 | 183 | 136 |
|  | Stage II | | 57 | 57 | 46 |
|  | Stage III | | 4 | 4 | 4 |
|  | Stage IV | | 2 | 2 | 2 |
| OLGIM | Stage 0 | | 458 | 451 | 369 |
|  | Stage I | | 96 | 94 | 76 |
|  | Stage II | | 17 | 17 | 15 |
|  | Stage III | | 13 | 13 | 10 |
|  | Stage IV | | 2 | 2 | 2 |
| Hp infection by Giemsa (number) | | |  |  |  |
| Antrum Hp-positive |  | | 257 | 249 | 180 |
| Fundus Hp-positive |  | | 234 | 227 | 163 |
| Both Hp-positive |  | | 215 | 208 | 154 |
| Either Hp-positive |  | | 276 | 268 | 189 |
| Both Hp-negative |  | | 310 | 309 | 283 |

IHC, immunohistochemistry; PCR, polymerase chain reaction; OLGA, Operative Link on Gastritis Assessment; OLGIM, Operative Link on Gastric Intestinal Metaplasia Assessment. *No significant differences (*p* > 0.05 for all comparisons) were observed between the two groups in clinicopathologic characteristics, including age, sex, ethnicity, symptoms, endoscopic atrophy (Kimura–Takemoto classification), histologic atrophy (OLGA/OLGIM stages), and *Helicobacter pylori* (Hp) infection status as determined by Giemsa staining.
