# Supplementary material for: Validation of the ABC Method for Gastric Cancer Risk Stratification Across Helicobacter pylori Infections With Diverse CagA Status and Subtypes in Brazil
Source: Cancer Med. 2025 Jun 27;14(13):e71016. doi: 10.1002/cam4.71016 (PMC12203232; doi:10.1002/cam4.71016)
Supplement: Supplementary file 5 — Table S2. Concordance between Helicobacter pylori classification by immunohistochemistry and polymerase chain reaction in 577 samples with successful immunohistochemical classification. [file CAM4-14-e71016-s006.docx]

**Supplementary Table S2:** Concordance between *Helicobacter pylori* classification by immunohistochemistry and polymerase chain reaction in 577 samples with successful immunohistochemical classification.

|  |  | Results by PCR | | | | |
| --- | --- | --- | --- | --- | --- | --- |
|  |  | Hp-negative | CagA-negative | Western-type | East Asian-type | Undetermined |
| Results by IHC | HP-negative | 283 | 10 | 2 | 0 | 7 |
|  | CagA-negative | 18 | 71 | 3 | 0 | 10 |
|  | Western-type | 3 | 6 | 100 | 0 | 42 |
|  | East Asian-type | 0 | 0 | 1 | 18 | 3 |

Similar to immunohistochemistry (IHC), *Helicobacter pylori* (Hp) infection status was categorized by polymerase chain reaction (PCR) as follows: Hp-negative (Hp(–)/CagA(–)/EPIYA-C(–)/EPIYA-D(–)); CagA-negative (Hp(+)/CagA(–)/EPIYA-C(–)/EPIYA-D(–)); Western-type (Hp(+)/CagA(+)/EPIYA-C(+)/EPIYA-D(–)); and East Asian-type (Hp(+)/CagA(+)/EPIYA-C(–)/EPIYA-D(+)). A total of 472 samples showed concordant classification by both IHC and PCR.
