# Supplementary material for: Validation of the ABC Method for Gastric Cancer Risk Stratification Across Helicobacter pylori Infections With Diverse CagA Status and Subtypes in Brazil
Source: Cancer Med. 2025 Jun 27;14(13):e71016. doi: 10.1002/cam4.71016 (PMC12203232; doi:10.1002/cam4.71016)
Supplement: Supplementary file 6 — Table S3. Comparison of gastric pathology scores and stages among 472 patients categorized by CagA status and subtype, based on concordant classification by immunohistochemistry and polymerase chain reaction. [file CAM4-14-e71016-s007.docx]

**Supplementary Table S3:** Comparison of gastric pathology scores and stages among 472 patients categorized by CagA status and subtype, based on concordant classification by immunohistochemistry and polymerase chain reaction.

|  |  | |  | CagA(+) versus CagA(–) infection | | | | | | | | | | | |  | East Asian-type versus Western-type  infection | | | | | | | | | | |
| --- | --- | --- | --- | --- | --- | --- | --- | --- | --- | --- | --- | --- | --- | --- | --- | --- | --- | --- | --- | --- | --- | --- | --- | --- | --- | --- | --- |
|  |  | |  | CagA (+) | CagA (–) | | | Odds  (95% CI) | | | | | *p* value* | | |  | East Asian | | | Western | | | Odds (95% CI) | | | *p* value* | |
| Antrum scores |  |  | | | | |  | | |  | |  | |  |  | | | | | |  |  | | |  | |  |
| Activity | ≥1 | |  | 108 | 60 | | | 2.0 | | | | | N.S. | | |  | 15 | | | 93 | | | 0.4 | | | N.S. | |
|  | 0 | |  | 10 | 11 | | | (0.7-5.5) | | | | |  | | |  | 3 | | | 7 | | | (0.1-2.5) | | |  | |
| Inflammation | ≥1 | |  | 116 | 70 | | | 0.8 | | | | | N.S. | | |  | 18 | | | 98 | | | ― | | | N.S. | |
|  | 0 | |  | 2 | 1 | | | (0.0-16) | | | | |  | | |  | 0 | | | 2 | | |  |  |  |  | |
| Atrophy | ≥1 | |  | 77 | 22 | | | 4.2 | | | | | <0.0001 | | |  | 13 | | | 64 | | | 1.5 | | | N.S. | |
|  | 0 | |  | 41 | 49 | | | (2.1-8.3) | | | | |  | | |  | 5 | | | 36 | | | (0.4-5.7) | | |  | |
| Metaplasia | ≥1 | |  | 39 | 4 | | | 8.2 | | | | | <0.0001 | | |  | 10 | | | 29 | | | 3.0 | | | N.S. | |
|  | 0 | |  | 79 | 67 | | | (2.8-33) | | | | |  | | |  | 8 | | | 71 | | | (1.0-9.8) | | |  | |
| Corpus scores |  |  | | | | |  | | |  | |  | |  |  | | | | | |  |  | | |  | |  |
| Activity | ≥1 | |  | 103 | 31 | | | 8.7 | | | | | <0.0001 | | |  | 17 | | | 86 | | | 2.8 | | | N.S. | |
|  | 0 | |  | 15 | 40 | | | (4.1-20) | | | | |  | | |  | 1 | | | 14 | | | (0.4-124) | | |  | |
| Inflammation | ≥1 | |  | 115 | 65 | | | 3.5 | | | | | N.S. | | |  | 18 | | | 97 | | | ― | | | N.S. | |
|  | 0 | |  | 3 | 6 | | | (0.7-22) | | | | |  | | |  | 0 | | | 3 | | |  |  |  |  | |
| Atrophy | ≥1 | |  | 44 | 9 | | | 4.1 | | | | | 0.0002 | | |  | 8 | | | 36 | | | 1.4 | | | N.S. | |
|  | 0 | |  | 74 | 62 | | | (1.8-10) | | | | |  | | |  | 10 | | | 64 | | | (0.4-4.4) | | |  | |
| Metaplasia | ≥1 | |  | 13 | 0 | | | ― | | | | | 0.0021 | | |  | 3 | | | 10 | | | 1.8 | | | N.S. | |
|  | 0 | |  | 105 | 71 | | |  |  |  |  |  |  | | |  | 15 | | | 90 | | | (0.3-8.1) | | |  | |
| Antrum & Corpus |  | | |  |  |  | | |  | |  | | | | | | |  |  | |  | | |  |  |  |  |
| OLGA stages | ≥I | |  | 90 | 26 | | | 5.5 | | | | | <0.0001 | | |  | 16 | | | 74 | | | 2.8 | | | N.S. | |
|  | 0 | |  | 28 | 45 | | | (2.8-11) | | | | |  | | |  | 2 | | | 26 | | | (0.6-27) | | |  | |
| OLGIM stages | ≥I | |  | 43 | 4 | | | 9.5 | | | | | <0.0001 | | |  | 11 | | | 32 | | | 3.3 | | | 0.0310 | |
|  | 0 | |  | 75 | 67 | | | (3.2-38) | | | | |  | | |  | 7 | | | 68 | | | (1.1-11) | | |  | |
|  |  | |  |  |  | | |  | | | | |  | | |  |  | | |  | | |  | | |  | |

Metaplasia, intestinal metaplasia; 95% CI, 95% confidence interval; N.S., not significant; OLGA, Operative Link on Gastritis Assessment; OLGIM, Operative Link on Gastric Intestinal Metaplasia Assessment. *Fisher’s exact test.
