# Supplementary material for: Validation of the ABC Method for Gastric Cancer Risk Stratification Across Helicobacter pylori Infections With Diverse CagA Status and Subtypes in Brazil
Source: Cancer Med. 2025 Jun 27;14(13):e71016. doi: 10.1002/cam4.71016 (PMC12203232; doi:10.1002/cam4.71016)
Supplement: Supplementary file 7 — Table S4. Comparison of gastric pathology (i) between East Asian‐type and Western‐type infections in Japanese Brazilians, and (ii) between Japanese and non‐Japanese Brazilians with Western‐type infection. [file CAM4-14-e71016-s008.docx]

**Supplementary Table S4:** Comparison of gastric pathology (i) between East Asian-type and Western-type infections in Japanese Brazilians, and (ii) between Japanese and non-Japanese Brazilians with Western-type infection.

|  |  |  | East Asian-type versus Western-type infection in Japanese Brazilians | | | |  | Japanese versus non-Japanese  Brazilians with Western-type infection | | | |
| --- | --- | --- | --- | --- | --- | --- | --- | --- | --- | --- | --- |
|  |  |  | East  Asian | Western | Odds (95% CI) | *p* value* |  | Japanese | Non-Japanese | Odds (95% CI) | *p* value* |
| Antrum & Corpus |  |  |  |  |  |  |  |  |  |  |  |
| OLGA stages | ≥I |  | 18 | 6 | 4.2 | N.S. |  | 6 | 102 | 0.8 | N.S. |
|  | 0 |  | 2 | 3 | (0.4-62.5) |  |  | 3 | 40 | (0.2-5.1) |  |
| OLGIM stages | ≥I |  | 13 | 4 | 2.3 | N.S. |  | 4 | 44 | 1.8 | N.S. |
|  | 0 |  | 7 | 5 | (0.4-15.6) |  |  | 5 | 98 | (0.3-8.7) |  |
|  |  |  |  |  |  |  |  |  |  |  |  |

95% CI, 95% confidence interval; N.S., not significant; OLGA, Operative Link on Gastritis Assessment; OLGIM, Operative Link on Gastric Intestinal Metaplasia Assessment. *Fisher’s exact test.
