# Supplementary material for: Validation of the ABC Method for Gastric Cancer Risk Stratification Across Helicobacter pylori Infections With Diverse CagA Status and Subtypes in Brazil
Source: Cancer Med. 2025 Jun 27;14(13):e71016. doi: 10.1002/cam4.71016 (PMC12203232; doi:10.1002/cam4.71016)
Supplement: Supplementary file 8 — Table S5. Correlation between serum markers and gastric pathology scores and stages in the 586 patients included in the study. [file CAM4-14-e71016-s001.docx]

**Supplementary Table S5:** Correlation between serum markers and gastric pathology scores and stages in the 586 patients included in the study.

Metaplasia, intestinal metaplasia; Hp, *Helicobacter pylori*; 95% CI, 95% confidence interval; N.S., not significant; OLGA, Operative Link on Gastritis Assessment; OLGIM, Operative Link on Gastric Intestinal Metaplasia Assessment; PG, pepsinogen. *Fisher’s exact test.

|  |  |  | HP antibody titer (U/mL) | | | |  | PGI level (ng/mL) | | | | |  | | PGII level (mg/mL) | | | | |  | | PGI/II ratio | | | | |
| --- | --- | --- | --- | --- | --- | --- | --- | --- | --- | --- | --- | --- | --- | --- | --- | --- | --- | --- | --- | --- | --- | --- | --- | --- | --- | --- |
|  |  |  | ≥5 | <5 | Odds  (95% CI) | *p* value* |  | ≤70 | >70 | Odds  (95% CI) | *p* value* |  | | ≥20 | | <20 | Odds  (95% CI) | *p* value* |  | | ≤3 | | >3 | Odds  (95% CI) | *p* value* |  |
| Antrum scores |  |  |  |  |  |  |  |  |  |  |  |  | |  | |  |  |  |  | |  | |  |  |  |  |
| Activity | ≥1 |  | 226 | 71 | 21 | <0.0001 |  | 165 | 229 | 0.7 | 0.0336 |  | | 143 | | 70 | 4.2 | <0.0001 |  | | 133 | | 55 | 4.9 | <0.0001 |  |
|  | 0 |  | 38 | 251 | (13-33) |  |  | 99 | 93 | (0.5-1.0) |  |  | | 121 | | 252 | (2.9-6.2) |  |  | | 131 | | 267 | (3.3-7.3) |  |  |
| Inflammation | ≥1 |  | 281 | 16 | 23 | <0.0001 |  | 261 | 133 | 0.7 | 0.0360 |  | | 182 | | 31 | 3.9 | <0.0001 |  | | 163 | | 25 | 4.2 | <0.0001 |  |
|  | 0 |  | 124 | 165 | (13-43) |  |  | 144 | 48 | (0.4-1.0) |  |  | | 223 | | 150 | (2.5-6.3) |  |  | | 242 | | 156 | (2.6-7.0) |  |  |
| Atrophy | ≥1 |  | 145 | 152 | 3.7 | <0.0001 |  | 136 | 258 | 1.0 | N.S. |  | | 88 | | 125 | 1.6 | 0.0149 |  | | 88 | | 100 | 2.1 | <0.0001 |  |
|  | 0 |  | 59 | 230 | (2.5-5.5) |  |  | 68 | 124 | (0.7-1.4) |  |  | | 116 | | 257 | (1.1-2.3) |  |  | | 116 | | 282 | (1.5-3.1) |  |  |
| Metaplasia | ≥1 |  | 70 | 227 | 2.0 | 0.0020 |  | 85 | 309 | 1.9 | 0.0091 |  | | 40 | | 173 | 1.0 | N.S. |  | | 51 | | 137 | 2.2 | 0.0004 |  |
|  | 0 |  | 39 | 250 | (1.3-3.1) |  |  | 24 | 168 | (1.2-3.3) |  |  | | 69 | | 304 | (0.6-1.6) |  |  | | 58 | | 340 | (1.4-3.4) |  |  |
| Corpus scores |  |  |  |  |  |  |  |  |  |  |  |  | |  | |  |  |  |  | |  | |  |  |  |  |
| Activity | ≥1 |  | 191 | 106 | 16 | <0.0001 |  | 128 | 266 | 0.5 | 0.0004 |  | | 143 | | 70 | 7.8 | <0.0001 |  | | 128 | | 60 | 7.1 | <0.0001 |  |
|  | 0 |  | 29 | 260 | (10-26) |  |  | 92 | 100 | (0.4-0.8) |  |  | | 77 | | 296 | (5.3-11.7) |  |  | | 92 | | 306 | (4.7-11) |  |  |
| Inflammation | ≥1 |  | 257 | 40 | 11.4 | <0.0001 |  | 237 | 157 | 0.8 | N.S. |  | | 168 | | 45 | 3.5 | <0.0001 |  | | 167 | | 21 | 8.3 | <0.0001 |  |
|  | 0 |  | 104 | 185 | (7.5-17) |  |  | 124 | 68 | (0.6-1.2) |  |  | | 193 | | 180 | (2.3-5.3) |  |  | | 194 | | 204 | (5.0-14) |  |  |
| Atrophy | ≥1 |  | 72 | 225 | 1.9 | 0.0034 |  | 85 | 309 | 1.6 | N.S. |  | | 53 | | 160 | 1.7 | 0.0167 |  | | 72 | | 116 | 5.2 | <0.0001 |  |
|  | 0 |  | 42 | 247 | (1.2-2.9) |  |  | 29 | 163 | (1.0-2.6) |  |  | | 61 | | 312 | (1.1-2.6) |  |  | | 42 | | 356 | (3.3-8.3) |  |  |
| Metaplasia | ≥1 |  | 16 | 281 | 0.7 | N.S. |  | 35 | 359 | 9.2 | 0.0001 |  | | 11 | | 202 | 0.7 | N.S. |  | | 30 | | 158 | 11 | <0.0001 |  |
|  | 0 |  | 21 | 268 | (0.4-1.5) |  |  | 2 | 190 | (2.3-80) |  |  | | 26 | | 347 | (0.3-1.6) |  |  | | 7 | | 391 | (4.4-29) |  |  |
| Antrum and Corpus |  |  |  |  |  |  |  |  |  |  |  |  | |  | |  |  |  |  | |  | |  |  |  |  |
| OLGA stages | ≥I |  | 172 | 125 | 3.7 | <0.0001 |  | 174 | 220 | 1.2 | N.S. |  | | 104 | | 109 | 1.5 | 0.0301 |  | | 121 | | 67 | 3.7 | <0.0001 |  |
|  | 0 |  | 79 | 210 | (2.6-5.3) |  |  | 77 | 115 | (0.8-1.7) |  |  | | 147 | | 226 | (1.0-2.1) |  |  | | 130 | | 268 | (2.5-5.5) |  |  |
| OLGIM stages | ≥I |  | 76 | 221 | 1.6 | 0.0280 |  | 102 | 292 | 2.2 | 0.0006 |  | | 45 | | 168 | 0.9 | N.S. |  | | 66 | | 122 | 2.9 | <0.0001 |  |
|  | 0 |  | 52 | 237 | (1.0-2.4) |  |  | 26 | 166 | (1.4-3.7) |  |  | | 83 | | 290 | (0.6-1.4) |  |  | | 62 | | 336 | (1.9-4.5) |  |  |
|  |  |  |  |  |  |  |  |  |  |  |  |  | |  | |  |  |  |  | |  | |  |  |  |  |
