# Supplementary material for: Validation of the ABC Method for Gastric Cancer Risk Stratification Across Helicobacter pylori Infections With Diverse CagA Status and Subtypes in Brazil
Source: Cancer Med. 2025 Jun 27;14(13):e71016. doi: 10.1002/cam4.71016 (PMC12203232; doi:10.1002/cam4.71016)
Supplement: Supplementary file 9 — Table S6. Performance of the ABC method for detecting OLGA and OLGIM stages in Japanese and non‐Japanese Brazilians. [file CAM4-14-e71016-s005.docx]

**Supplementary Table S6**: Performance of the ABC method for detecting OLGA and OLGIM stages in Japanese and non-Japanese Brazilians.

|  | Either pepsinogen or antibody test in the ABC method | | | | | | | | | | | | | | | | | | | |
| --- | --- | --- | --- | --- | --- | --- | --- | --- | --- | --- | --- | --- | --- | --- | --- | --- | --- | --- | --- | --- |
|  | Japanese Brazilians | | | | |  | Non-Japanese Brazilians | | | | | |  | | Both (Total cases) | | | | | |
|  | positive B/C/D | negative A |  | positive B/C/D | negative A |  | positive B/C/D | negative A |  | positive B/C/D | negative A |  | | positive B/C/D | | negative A |  | positive B/C/D | negative A |  |
| For OLGA/OLGIM stages | OLGA | |  | OLGIM | |  | OLGA | |  | OLGIM | |  | | OLGA | | |  | OLGIM | |  |
| For detecting stages ≥ I |  | |  |  | |  |  | |  |  | |  | |  | | |  |  | |  |
| Stages I, II, III, and IV (n) | 33 | 21 |  | 26 | 16 |  | 159 | 38 |  | 68 | 18 |  | | 192 | | 59 |  | 94 | 34 |  |
| Stage 0 (n) | 18 | 53 |  | 25 | 58 |  | 130 | 134 |  | 221 | 154 |  | | 148 | | 187 |  | 246 | 212 |  |
| *p* value* | 0.0001 | |  | 0.0010 | |  | <0.0001 | |  | 0.0005 | |  | | <0.0001 | | |  | 0.0001 | |  |
| Sensitivity (%) | 61 | |  | 62 | |  | 81 | |  | 79 | |  | | 76 | | |  | 73 | |  |
| Specificity (%) | 75 | |  | 70 | |  | 51 | |  | 41 | |  | | 56 | | |  | 46 | |  |
| PPV (%) | 65 | |  | 51 | |  | 55 | |  | 24 | |  | | 56 | | |  | 28 | |  |
| NPV (%) | 72 | |  | 78 | |  | 78 | |  | 90 | |  | | 76 | | |  | 86 | |  |
|  |  |  |  |  |  |  |  |  |  |  |  |  | |  | |  |  |  |  |  |
| For detecting stages ≥ II |  |  |  |  |  |  |  |  |  |  |  |  | |  | |  |  |  |  |  |
| Stages II, III, and IV (n) | 15 | 5 |  | 8 | 5 |  | 40 | 3 |  | 17 | 2 |  | | 55 | | 8 |  | 25 | 7 |  |
| Stage 0 and I (n) | 36 | 69 |  | 43 | 69 |  | 249 | 169 |  | 272 | 170 |  | | 285 | | 238 |  | 315 | 239 |  |
| *p* value* | 0.0010 | |  | N.S. | |  | <0.0001 | |  | 0.0141 | |  | | <0.0001 | | |  | 0.0256 | |  |
| Sensitivity (%) | 75 | |  | 62 | |  | 93 | |  | 89 | |  | | 87 | | |  | 78 | |  |
| Specificity (%) | 66 | |  | 62 | |  | 40 | |  | 38 | |  | | 46 | | |  | 43 | |  |
| PPV (%) | 29 | |  | 16 | |  | 14 | |  | 6 | |  | | 16 | | |  | 7 | |  |
| NPV (%) | 93 | |  | 93 | |  | 98 | |  | 99 | |  | | 97 | | |  | 97 | |  |
|  |  |  |  |  |  |  |  |  |  |  |  |  | |  | |  |  |  |  |  |
| For detecting stages ≥ III |  |  |  |  |  |  |  |  |  |  |  |  | |  | |  |  |  |  |  |
| Stages III and IV (n) | 2 | 0 |  | 4 | 3 |  | 3 | 1 |  | 7 | 1 |  | | 5 | | 1 |  | 11 | 4 |  |
| Stage 0, I, and II (n) | 49 | 74 |  | 47 | 71 |  | 286 | 171 |  | 282 | 171 |  | | 335 | | 245 |  | 329 | 242 |  |
| *p* value* | N.S. | |  | N.S. | |  | N.S. | |  | N.S. | |  | | N.S. | | |  | N.S. | |  |
| Sensitivity (%) | 100 | |  | 57 | |  | 75 | |  | 88 | |  | | 83 | | |  | 73 | |  |
| Specificity (%) | 60 | |  | 60 | |  | 37 | |  | 38 | |  | | 42 | | |  | 42 | |  |
| PPV (%) | 4 | |  | 8 | |  | 1 | |  | 2 | |  | | 1 | | |  | 3 | |  |
| NPV (%) | 100 | |  | 96 | |  | 99 | |  | 99 | |  | | 100 | | |  | 98 | |  |

Groups B/C/D, combined Groups B, C, and D (i.e., groups other than Group A), for the analysis; OLGA, Operative Link on Gastritis Assessment; OLGIM, Operative Link on Gastric Intestinal Metaplasia Assessment; PPV, positive predictive value; NPV, negative predictive value; N.S., not significant. *Fisher’s exact test.
